# Unravelling the art of developing skilled communication: a longitudinal qualitative research study in general practice training

**DOI:** 10.1007/s10459-024-10403-6

**Published:** 2024-12-17

**Authors:** Michelle Verheijden, Angelique Timmerman, Dorien de Buck, Anique de Bruin, Valerie van den Eertwegh, Sandra van Dulmen, Geurt T. J. M. Essers, Cees van der Vleuten, Esther Giroldi

**Affiliations:** 1 Present Address: Department of Family Medicine, Care and Public Health Research Institute (CAPHRI), Maastricht University, Maastricht, The Netherlands; 2 Department of Educational Development and Research, School of Health Professions Education (SHE), Maastricht University, Maastricht, The Netherlands; 3 Department of Primary and Community Care, Radboud University Medical Center, Family, Nijmegen, The Netherlands; 4 Department of Skillslab, Faculty of Health, School of Health Professions Education (SHE), Maastricht University, Maastricht, The Netherlands; 5 Netherlands Institute for Health Services Research (NIVEL), Utrecht, Netherlands; 6 Department of Primary and Community Care, Radboud University Medical Center, Nijmegen, Netherlands; 7 Faculty of Caring Science, Work Life and Social Welfare, University of Borås, Borås, Sweden; 8 Independent Researcher, Utrecht, The Netherlands

**Keywords:** Longitudinal qualitative research, Medical training, Workplace learning, Doctor-patient communication, Skilled communication

## Abstract

Doctor-patient communication is a core competency in medical education, which requires learners to adapt their communication flexibly to each clinical encounter. Although conceptual learning models exist, information about how skilled communication develops over time is scant. This study aims to unpack this process of communication learning and to identify its facilitators. We conducted a longitudinal qualitative study employing a constructivist grounded theory approach in a General Practice training setting. Over a 6-month period, we closely monitored 8 first-year and 5 third-year trainees (n = 13) by means of clinical observations, stimulated recall interviews and audio diaries. In an iterative process of data collection and analysis, we triangulated these sources across trainees (horizontal analysis) and over time (vertical analysis) to identify how themes evolved. This analysis led us to the construction of a six-stage cyclic conceptual model during which trainees: (1) have an impactful experience; (2) become aware of own communication; (3) look for alternative communication behaviours; (4) experiment with new behaviours; (5) evaluate the effectiveness; and (6) internalise the new communication behaviours. Additionally, conditions were found to support learning and reflection. Becoming a skilled communicator seems to require a continuous approach, with repeated practice and reflection to adapt and internalise communication in the clinical encounter. Consequently, we recommend that trainees be supported with tailored feedback to strengthen their communication repertoire. Ideally, they should be guided by supervisors who serve as coaches in a safe learning environment with regular, planned learning activities.

**Supplementary Information:**

The online version contains supplementary material available at 10.1007/s10459-024-10403-6.

## Introduction

Doctor-patient communication is a core competency in medical education (Frank, 2015; (AcfGME, 2024)). Medical schools have long recognised its importance, integrating generic communication skills training into their curricula (Deveugele et al., 2005; Silverman, 2011). As recent studies have emphasised, learning communication involves more than simply completing checklists of generic communication skills (Giroldi et al., 2017; Salmon & Young, 2011; Zoppi & Epstein, 2002). Rather, it requires flexibility and creativity to adapt communication to the needs of each individual patient (Salmon & Young, 2011; Verheijden et al., 2023b). This calls for a contextualised approach that enables learners to adapt communication flexibly to the clinical encounter, referred to as ‘skilled communication’ (Salmon & Young, 2011).

Previous studies have defined learning skilled communication as a process that interacts closely with the clinical context, often triggered by impactful experiences or concrete stimuli (van den Eertwegh et al., 2015; Giroldi et al., 2017). By developing conceptual models that visualise trainees’ learning process and strategies, these studies have offered insights into how learners become skilled communicators during workplace learning. Both van den Eertwegh et al. (2015) and Giroldi et al. (2017) explored this process in General Practitioner (GP) trainees, developing conceptual learning models with overlapping learning stages. In both models, learning begins with a stimulus or impactful experience and continues as trainees reflect on the communication strategies used. Trainees then experiment with alternative behaviours, refining these strategies through ongoing practice and feedback until they develop a personalised communication style. These models emphasize the role of repeated practice and reflection to integrate communication behaviours into the learners’ personal repertoire (Giroldi et al., 2017; van den Eertwegh et al., 2015). While these models complement each other, van den Eertwegh’s model places more emphasis on developing awareness and personalising communication through trial-and-error to personalise communication behaviours. In contrast, Giroldi’s model explicitly highlights the learning stages of identifying and evaluating communication strategies before mastery is reached.

To develop these models, van den Eertwegh et al. (2015) primarily observed GP trainees in clinical practice and conducted interviews, whereas Gioldi et al. (2017) relied on focus groups and interviews involving both GP trainees and their supervisors. Both studies used self-reported data, like focus groups and interviews, to explore how trainees learn communication in the workplace (van den Eertwegh et al., 2015; Giroldi et al., 2017). To our knowledge, no studies (van den Eertwegh et al., 2015; Giroldi et al., 2017) have combined self-reported data with naturalistic data, such as observations of clinical encounters, which could provide deeper insights into the real-time complexities and contextual nuances of learning skilled communication. Using naturalistic data is crucial, as it captures trainees’ actual communication behaviours, providing a more detailed picture of how skilled communication is developed. In addition, it remains unknown if the learning strategies described in the aforementioned conceptual learning models are actually observed and used in educational practice.

Furthermore, our understanding of how learning of skilled communication evolves *over* time remains limited (van den Eertwegh et al., 2015; Giroldi et al., 2017). Prior studies (Giroldi et al., 2017; van den Eertwegh et al., 2015) have found that learning skilled communication is a long-term process (Kennedy et al., 2005; Schuwirth & van der Vleuten, 2006), however they have often been based on cross-sectional data (Giroldi et al., 2017; van den Eertwegh et al., 2015), that only reflect learning at a single point in time. A longitudinal lens might shed more light on how a complex skill such as communication evolves in workplace learning (Balmer et al., 2021; Zacharias et al., 2002), which often happens informally and spontaneously through practice (Eraut, 2004). As Swanwick (2005) suggests, this process is implicit, making it difficult to be recognised as learning through single-time-point studies (Swanwick, 2005). Understanding how learners develop expertise over time can help identify the learning process and conditions that facilitate this development (Balmer et al., 2021). Teunissen further emphasizes that cognitive and socio-cultural theoretical perspectives are interconnected. By understanding both, we could enhance learning to become skilled communicators over time (Teunissen, 2010).

By exploring how learners become skilled communicators over time in the clinical workplace, we might gain more insights into how communication skills are applied across various clinical contexts. This underscores the need for a longitudinal approach to understand how training can best support the transfer of communication learning to clinical practice (van den Eertwegh et al., 2015). Indeed, the question of how learners can apply learned communication skills in clinical practice is becoming more pertinent (Hawken, 2005; van Weel-Baumgarten, 2016). Prior research in undergraduate medical education has demonstrated that isolated communication skills training does not promote the effective transfer of skills into clinical practice (Lie et al., 2010; van Dalen et al., 2002; Zimmermann et al., 2021).

The present study builds upon previous research (van den Eertwegh et al., 2015; Giroldi et al., 2017) to enrich our understanding of how learning of communication actually develops and transfers to clinical practice. We therefore aimed to explore the longitudinal process of skilled communication in real-life practice, alongside the conditions that support this process. Our findings may offer recommendations on how best to support learners in becoming skilled communicators.

## Methods

### Study design

We conducted an explorative study using a constructivist grounded theory approach (Audulv et al., 2022; Charmaz, 2017; Glaser & Strauss, 2017; Reeves et al., 2013). The aim of this research method is to understand a phenomenon’s social process and dynamics by generating an empirical theory that is grounded in the data (Audulv et al., 2022). We deemed this approach suitable to unravel trainees’ communication learning (Charmaz, 2017) as it allowed us to explain social processes by generating new concepts that map participants’ understandings, strategies and the relationships between them (Strauss, 1987). In order to unpack trainees’ learning process, we employed a longitudinal study design spanning six months. It also allowed us to explore how trainees’ individual learning evolved over time, yielding unique stories about their learning experiences.

### Study context

The study was set in a General Practice (GP) specialty training. Sampling from two out of eight training institutes in the Netherlands (Maastricht and Nijmegen), we chose this setting because of its contextual variability: GP trainees have encounters with a varied population and therefore have to deal with a host of conditions, including acute and chronic diseases. Consequently, they often build long-standing and intensive relationships with their patients (Essers et al., 2011; Svenberg et al., 2007; Veldhuijzen et al., 2013). The GP training programme spans three years in total; the first and third year consist of workplace learning in a GP practice. The second year provides experience in multiple hospital-based residencies, such as the emergency department. Throughout the training programme, four days of workplace learning are alternated with one day of education at the training institute. The weekly education day at the training institute includes supervision by both a GP and a behavioural scientist, focusing on developing trainees’ medical knowledge, communication skills, and developing attitudinal aspects regarding patient-centeredness. During these education days, GP trainees primarily engage in peer group learning with fellow GP trainees. Trainees self-regulate their learning through gathering feedback, reflection and formulating learning goals and personal plans. Besides, learning in small peer groups, assessment of communication takes place on an individual basis and includes a formative consultation test in which an independent trained GP assessor uses a validated behaviour rating scale and writes a narrative feedback report about the consultation performance of the trainee. In the workplace, GP trainees are supervised by an experienced GP who regularly provides on-the-job feedback. Trainees are often observed by their supervisor during weekly (joint) consultation hours and engage in reflective learning conversations with their supervisor to review a videotaped consultation.

### Participant recruitment

In qualitative longitudinal research, sample sizes are routinely small, ranging from 6 to 29 participants (Balmer et al., 2021; Smithbattle et al., 2018). In order to obtain a varied sample in terms of trainee gender, practice setting and patient mix, we aimed to recruit 10–15 trainees. We did so by means of purposive sampling on the aforementioned weekly educational release days. With the consent of trainees’ teachers, we approached 11 groups of first- and third year trainees to provide information about the study purpose, procedure and required time investment. Trainees who were willing to participate were contacted individually to obtain their informed consent. Once granted, trainees completed a questionnaire on demographic information before participation. We did not approach any second-year trainees, as their residencies do not take place in a GP practice.

### Data collection

**Fig. 1:**
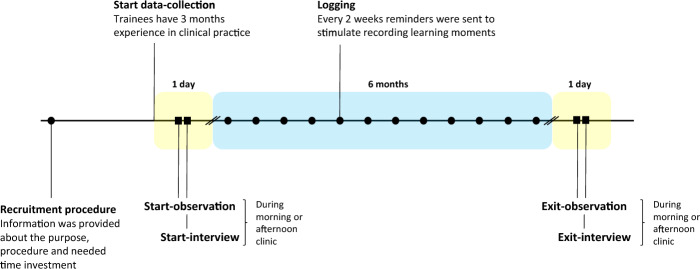
Timeline of data-collection process* *Time-points on timeline: Recruitment procedure: Information was provided about the purpose, procedure and needed time investment. Start-data-collection: Data-collection started when ttrainees had three months experience in a general practice. Start-observation and start-interview. Logging: Every 2 weeks reminders were sent to stimulate trainees to record learning moments for their audio diary. Exit-observation and exit-interview

Data were collected (Fig. 1) in the period spanning September 2021 to June 2022 by means of clinical observations, stimulated-recall interviews and audio diaries. The observations and interviews took place at two points in time, with a six-month interval during which time each trainee composed an audio diary (Calman et al., 2013; Dubé et al., 2015). To afford trainees the time to acclimatise in their GP practice, we initiated data collection from the third month of their training year onwards.

The observations of patient encounters took place during a regular morning or afternoon clinic and were audiotaped with patients’ prior consent. To obtain their consent, the participating trainees first explained verbally to each individual patient, emphasising that the research focused on trainees’ communication during the clinical encounter. During the observations, we made field notes of salient doctor-patient communication. The assembled data served as input to the interview (hereafter referred to as start interview) that followed upon the first observation round. After six-month interval, the trainees were visited again in their practice for a second observation and a follow-up interview (hereafter referred to as exit interview). Both interviews used the method of stimulated reflection and recursive interviewing (Calderhead, 1981; Hermanowicz, 2013). More specifically, the start interview (Appendix A) stimulated reflection on trainees’ learning, their strategies and potential factors that supported or hindered their learning. In the exit interview (Appendix B), trainees were invited to reflect on their experiences and strategies of the past period to elucidate how these shaped their present learning. We also used their audio diaries to explore how trainees shaped their learning processes between the start -and exit interview.

We asked trainees to audio-record communication learning moments using a privacy-compliant smartphone application (GDPR, E-privacy, NHS information governance, NEN, DCB 0129, ICO, and ISO-27001). We chose for this data collection format, as it provides an opportunity to select and narrate ongoing learning moments close to the time of those events (Atherley et al., 2023; Monrouxe, 2010). This allowed trainees to more easily recall and share their experiences during these events. We chose audio diaries over written diaries as they are less time-consuming and are likely to be considered as less of a burden, to encourage completion after a long day in clinical practice (Fisher & Noble, 2004). We instructed trainees to audio-record approximately one learning moment every two weeks and sent them reminders to stimulate recording (García et al., 2016; Gordon et al., 2017). To support them, we also provided a guideline including audio diary prompts to stimulate reflection (Verma, 2021) and instructions on how to use the smartphone application (Appendix C).

### Data analysis

Before data analysis, all data were transcribed verbatim. The researchers read all transcripts and listened to all recordings, to become familiar with the content of the interviews, field-notes of the clinical observations and audio diaries. We used a thematic content analysis with a constant comparative approach to identify and categorise overarching themes in the data (Bowen, 2006; Holton, 2010), intending to expand current conceptual models (van den Eertwegh et al., 2015; Giroldi et al., 2017) and relative methodological constructs of longitudinal qualitative research (Balmer et al., 2021; Stephens et al., 2019; Zacharias et al., 2002), using grounded theory (Charmaz, 2006, 2017).

First, to maximise thematic variability (Kiger & Varpio, 2020; Stephens et al., 2019), we treated the interviews, observations and audio diaries as separate datasets. Three members of the research team (MV, EG, AT), including three medical students (DB, LV, LeS), carried out line-by-line coding of the data in pairs by formulating general descriptions which resulted in codes (Balmer et al., 2021; Holton, 2010; Zacharias et al., 2002). Consistent with a constructivist stance, sensitising concepts drawn from relevant theoretical constructs described in the introduction section, guided this synchronic analysis (Charmaz, 2006; Glaser & Strauss, 2017; Johnson et al., 2023). Second, the researchers discussed and re-examined any discrepancies in interpretations in these identified general descriptions (i.e., codes) until they reached consensus on their content, resulting in categories for subsets of data. To ensure the coding reliability, we provided category descriptions and developed a comprehensive code book (Appendix D). Third, as data analysis progressed, we merged the interview, observation and audio diary data, thereby allowing both horizontal and vertical triangulation (Balmer et al., 2021; Mays & Pope, 2000; Tavakol & Sandars, 2014). More specifically, we performed a vertical analysis by grouping categories into overarching themes to establish relationships across all participant data. In this way, we gained more information on a ‘collective level’. In the horizontal analysis, on the other hand, we focused on comparing the overarching themes within the data over time for the groups of first- and third year trainees, to enhance our understanding of how trainees integrated communication behaviours and learning strategies into their learning and how it evolved (Gordon et al., 2017; Hermanowicz, 2013; Smithbattle et al., 2018). To gain insight in longitudinal learning during the training programme, this analysis compared the development of communication performance during the first and third year of training. Finally, to deepen our analysis, the research team reflected on the internal coherence of findings allowing to identify connections in the data, resulting in a conceptual learning model of communication that applied to all trainees. NVivo software, version 12 Pro (2018, QSR International), was employed to manage the data analysis.

### Reflexivity

Throughout data collection, analysis, and writing up the results, our research team maintained reflexivity by discussing knowledge, personal experiences and views on doctor-patient communication and workplace-based learning (Atherley et al., 2023). Team members had diverse academic backgrounds and experiences: MV is a PhD candidate and GP trainee; AT is a psychotherapist, communication trainer, curriculum designer and researcher with experience in mixed-methods research designs; EG is a health scientist and researcher versed in qualitative research in the field of communication; and AdB is an educational and cognitive psychologist with a strong record of research in self-regulated learning. This combination of backgrounds and interests led to repeated discussion on how team members’ beliefs affected their analysis and interpretation of the data.

As a GP trainee and a researcher, the principal researcher (MV) served as both an ‘insider’ with shared experiences and an ‘outsider’ who did not assess participants learning (Dwyer & Buckle, 2009; Mercer, 2007). On the one hand, the close relationship that the principal researcher developed with participants, arising from shared experiences as a trainee, may have created a supportive environment in which trainees felt comfortable to share learning experiences (A. Atherley et al., 2021; Smithbattle et al., 2018). On the other hand, this closeness could have potentially imposed pressure on participants to participate, possibly leading to less authenticity in their responses. Furthermore, the principal researcher’s experiences might have induced a lack of objectivity, in turn influencing data collection and analysis. To counter this influence in writing up the results, the principal researcher kept a logbook in which she reflected on how her personal experiences and pre-constructed knowledge could have influenced the interpretation of the data. Her reflections touched upon how her interactions with patients, her presence during the consultations and her beliefs about the construction of knowledge through interactions affected her perception of the data. To enhance trustworthiness, clinical observations were conducted in pairs, with the principal researcher collaborating with at least one researcher (AT, EG) or a medical student (DB, LV, LeS). She also discussed her reflections within the research team (AT, EG) and the aforementioned medical students who also analysed the data.

Ultimately, the process of data-collection, analysis, and results write up significantly influenced the development of the principal researcher’s communication learning process. The shared experiences and challenges encountered by participants in this study fostered her own awareness of communication behaviour. This awareness prompted her to experiment with new behaviours and evaluate their effectiveness. For example, she learned from her peers that regular planned joint consultation hours were beneficial for learning, particularly when focusing on specific communication behaviours like setting boundaries.

### Ethics approval

We obtained ethics approval from the Ethics Review Committee of the Faculty of Health, Medicine and Life Sciences (FHML-REC) at Maastricht University (file no. FHML-REC-2021/039). All participants (i.e., GP trainees, clinical supervisors and patients present during the observations) gave their informed consent prior to the start of the data collection. Participation was voluntary and trainees received a 50-euro gift voucher to compensate for their time investment. We replaced all information in the manuscript that could disclose participants’ identity by one or more artificial identifiers (pseudonyms).

## Results

In the following paragraphs, we will first describe the demographic composition of our participant group, before addressing our research question by outlining a conceptual model in which we identified learning strategies. Next, we will delve into the stages of learning and the conditions that supported trainees in becoming skilled communicators.

### Data characteristics

Over a 6-month period, we conducted 26 interviews with a total of 13 participants, yielding 115 audio diaries with a total of 542.68 recorded minutes (approximately 9 h). Approximately nine audio diaries per participant were collected with a mean duration of 41, 75 min in total (SD 27.9 min). Interviews lasted between 60 to 90 min. Eight first-year (61%) and five third-year (39%) GP trainees participated in this study. The trainees were aged between 28 and 35 years (mean: 30 years; SD: 1.7), four trainees were male (31%) and all had previous work experience in different medical specialties (see Appendix E).

### Conceptual model of learning skilled communication in the workplace

**Fig. 2:**
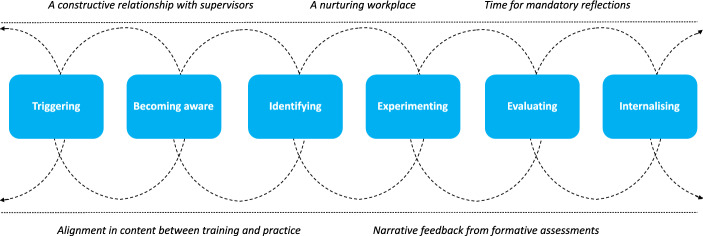
A six-stage conceptual communication learning model incorporating learning strategies and supporting conditions*. *The dotted spiral line in this model depicts the diverse pathways that trainees pass through, moving back and forth through the various stages with arrows signalling various possible routes. The dotted line in this model creates a natural transition to the conditions that support trainees learning

Our scrutiny of the data led to the identification and construction of a six-stage cyclic conceptual model of learning that may characterise the learning process and the conditions that support trainees in developing skilled communication. The model outlines stages of learning, which represent trainees’ progression, and identified various supportive conditions that facilitate this development. The model is outlined in Fig. 2 and the terms used are defined in Box 2 (Appendix F). According to this model, trainees (1) have an impactful experience or concrete stimulus; (2) become aware of own communication; (3) look for alternative communication behaviours; (4) experiment with new behaviours; (5) evaluate the effectiveness, and (6) internalise the new communication behaviours. In the conceptual model, trainees could pass the learning stages through diverse pathways.

### Learning of skilled communication over time

Below, we will describe each stage of the conceptual model, focusing on the longitudinal development of both first-and third-year trainees and how they approach their learning. The outlined stages summarize in a narrative format common learning strategies used during the first and third year of the training programme while also highlighting subtle differences in how trainees at each stage apply these strategies. If the results concern either the group of first- or third year trainees, we will outline this, to explicate differences in how during different stages of training the focus of communication learning differs and what supported learning.

#### Stage 1—The trainee has an impactful experience

Learning frequently started with experiencing an impactful experience that challenges trainees’ communication behaviours. These experiences often motivated them to seek improvement in their communication behaviours. Throughout their training, both the group of first- and third-year trainees encountered impactful experiences when faced with communication challenges or evoked emotions such as frustration or discomfort. These impactful experiences, often occurring during or after clinical encounters, are pivotal in initiating the learning process. To render an experience impactful and start the learning process, a few of the group of first- and third-year trainees took time for grading appropriateness of one’s own thoughts and communication behaviours (self-judgement). Taking time for self-judgement happened either immediately after clinical encounters or when watching videotaped consultations with peers or supervisors, which enhanced their self-monitoring practices.

In the start interviews, first-year trainees often mentioned challenges such as managing escalating situations with patients and adhere to time management during encounters. By the exit interviews, focus in challenges for first-year trainees had shifted to how to structure consultations with patients who presented psychological, rather than somatic, symptoms. First-year trainees used concrete feedback from supervisors on communication behaviour as a key stimulus and spark reflection. Third-year trainees experienced uneasiness when they had difficulty to establish a connection with patients or when they felt unable to meet patients’ expectations. Supervisors encouraged them to review their communication behaviour, particularly when they doubted whether they had fully met patients’ needs or requests for medical care.

‘It’s a feeling that I pick up. I think it’s very difficult to describe, what it is that I notice but most of all it’s those doubts or that feeling that you have that you don’t fully meet their expectations or what they would like’ (GP trainee 1004; start interview).

By the exit interviews, most of the group of third-year trainees relied on observations of their peers’ communication behaviours through videotaped consultations on educational days as an impactful experience triggering learning.

#### Stage 2—The trainee becomes aware of own communication

Impactful experiences seemed to raise trainees’ awareness of their communication behaviours. Through reflection, they gained a deeper understanding of how and why they had used certain communication behaviours. During this stage, trainees acknowledged that reflecting on impactful experiences with their supervisors and peers helped raise awareness of communication behaviours. These discussions helped make their impactful experiences more explicit, enhancing their understanding of how they applied communication behaviours in clinical practice. Informal discussions with peers outside training sessions also contributed to this awareness. Over time, self-reflection was reinforced by external feedback from supervisors and peers. Both the group of first- and third-year trainees consistently reported in interviews and audio diaries that discussing videotaped consultations was especially valuable for raising awareness, with their supervisors often asking reflective questions to stimulate deeper thought. Trainees also used self-monitoring strategies to become aware of their communication behaviour, asking themselves questions like: ‘Did it go the way I wanted?’, ‘Could I have handled it differently?’ Some first-and third-year trainees noted that this awareness did not always emerge immediately after consultations, but rather later, at home, when they experienced more mental space to reflect on their communication behaviour.

Third-year trainees were also prompted by their supervisors The following extract from a first-year trainee’s audio diary clearly demonstrates such awareness:

‘At the moment I am watching my own videotaped consultations again. There are a couple of things that struck me when I look at myself. I would like to talk about that [during this audio-diary]. I noticed that I quite often have my arms crossed during the consultation. So, they [teacher/supervisor] always say that it can come across as very uncommunicative’ (GP trainee 1006; audio diary).

Some first-years trainees also used communication interaction models, such as the Rose of Leary, and posed reflective questions to themselves. During the start interviews, supervisors also prompted third-year trainees to deliberately reflect on their communication behaviour with reflective questions like: ‘Why did you choose to interrupt the patient when taking their history?’ Similarly, observing supervisors and receiving their feedback during joint consultation hours increased trainees’ awareness of their communication behaviours. This was mentioned by some first-year trainees during the exit interviews and some third-year trainees during the start interviews. For supervisor feedback to result in behavioural change, however, some of the group of third-year trainees emphasized the need to personally recognise the suggested points for improvement. These third-year trainees also noted in their audio diaries that observing their colleagues during clinical encounters and seeking feedback from peers, family, or friends could equally boosted their awareness. Unlike their first-year peers, they also flagged formal training moments such as programmed assessments (through the use of videotaped consultations) as important to reach awareness.

#### Stage 3—The trainee looks for alternative communication behaviours

Once trainees had become aware of their communication behaviour, they were motivated to find alternative communication behaviours, such as taking back the lead in consultations with talkative patients. All trainees consistently relied on their supervisors for advice on which strategies to use. Supervisors guided the trainees by suggesting concrete sentences to adapt their communication behaviour. Also, by posing reflective questions, supervisors encouraged their trainees to explore alternative communication behaviours, especially during discussions of videotaped consultations. It was important that alternative communication behaviours aligned with trainees’ communication style. From the start interviews with first-year trainees we understood that joint consultation hours with supervisors were a primary source of inspiration for such alternative communication behaviours:

‘…we [me and my supervisor] do consultation hours together, so she [supervisor] can see things happen. Then she often goes like ‘you could’ve done it like this’ or ‘I might handle it like that’. We also take turns, so I also get to see how she does it. That also helps’ (GP trainee 1012; exit interview).

By the exit interviews, first-year trainees indicated that feedback obtained from peers during communication training sessions had a more significant impact than that of supervisors. Furthermore, a learning strategy used by a few first-year trainees was to verbalise their thought processes regarding how to approach similar future communication situations differently. In audio diaries they also reported that tools such as a standardised consultation structure or concrete sentences learnt in communication training helped them to identify alternative communication behaviours.

Most of the group of third-year trainees indicated in start interviews that peers played an important role in providing feedback on how to handle specific communication situations differently:

‘Yes, certainly, for instance when someone else says something I hadn’t considered yet. When someone [peer] says ‘yeah, I always say this’ or ‘I always say that’, oh right, maybe that can help. Very well, I should try that once’ (GP trainee 1011; start interview).

In fact, by the time of the exit interviews, most of these third-year trainees drew more inspiration from observations or discussions with colleagues and peers than from supervisor feedback.

#### Stage 4—The trainee experiments with new communication behaviour

As a next step, all trainees started experimenting with the new communication behaviours previously found, by transferring them to the clinical encounter. They often focused on specific communication behaviours, such as setting boundaries during consultations using concrete sentences learnt in the previous stage. This remained their focus all the way through to the exit interviews. For example, when patients presented multiple complaints, trainees would point to the time constraints of the consultation and advise them to book a follow-up appointment.

Most first-year trainees tended to experiment with new ways to identify patients’ reasons for their visit and to structure the consultation. By the exit interviews, first-year trainees emphasised the importance of stepping out of their comfort zones when experimenting with new communication behaviours. However uncomfortable they were at first, with encouragements from their supervisors they felt less stressed to take the leap. In the ensuing encounter, a first-year trainee experienced that experimenting with new communication behaviour resulted in a not foreseen outcome:

‘You do need to gather experience. For instance, I once had a situation or, rather, I got the tip that I should try to say a bit louder whether or not something is normal, right? Instead of a little bit louder, right, like that, and then I also landed in trouble because I said it in a situation where it wasn’t very wise to say it that loud. So afterwards I thought to myself, well, I had better not have used that here now’ (GP trainee 1013; start interview).

Third-year trainees often experimented with new communication behaviours to collaborate with patients who insisted on referrals or medication prescriptions and to maintain a balance between taking the lead and addressing patients’ agenda:

‘Well, for instance by developing a specific communication behaviour. This was a lady [patient] who was quite insistent. But by using certain ways of boundary setting, you give her less space. So that is what I try to do, to see if I can improve my boundary settings during the consultation’ (GP trainee 1002; start interview).

To fully master the new communication behaviours, third-year trainees frequently emphasised the importance of practicing them repeatedly in similar situations during encounters. Nevertheless, the group of third-year trainees noted that they sometimes missed opportunities to experiment, for they did not always know in which situations they could best do so. All third-year trainees stressed that being mindful of a new communication behaviour before the clinical encounter was paramount. It was perceived essential to remain their focus during a clinical encounter when experimenting with new behaviours to avoid reverting to old habits.

#### Stage 5—The trainee evaluates the new communication behaviour

The previous stage of experimentation naturally led to an evaluation of the new communication behaviours. Both the group of first- and third-year trainees evaluated these behaviours by using patient feedback, self-reflection, and personal feelings about the appropriateness and effectiveness of their communication behaviours in various clinical contexts. Over time, both first- and third-year trainees grow more confident in their ability to evaluate their communication behaviours. They begin integrating patient feedback and personal feelings into a more intuitive process, using tools like supervisor feedback and structured assessments.

More specifically, first-year trainees often sought explicit feedback from patients by directly asking them how they had experienced the encounter and whether they were satisfied. Some of them also used physical signals to evaluate their behaviours. For instance, they took a dry mouth for a sign of excessive talking, which undermined patient engagement. Many first-year trainees also recorded in their audio diaries that they aimed to identify moments of success in the clinical encounter. These moments were when they felt their new communication behaviours were effective or time-efficient, such as refraining from interrupting the patient too early or using humour to comfort patients. Supervisor feedback and formal assessment tools, like the MAAS-global (Thiel et al., 2000), were also important evaluation tools for them. By the time of the exit interviews, their ability to assess their emotions and understand how these emotions impacted their communication behaviour had progressed, boosting their confidence. As one first-year trainee noted:

‘I think to myself at that point like, is it appropriate for this specific point in the consultation? Or I think to myself, that is not how I want to be as a doctor. So, in that case it is more about how I think a doctor should be’ (GP trainee 1006; exit interview).

Third-year trainees mostly tend to focus on patients’ verbal and non-verbal responses in relation to their own feelings to evaluate the effectiveness of their communication behaviours:

‘I noticed that I actually don’t get any unpleasant responses from the patients at all. It is actually going quite well. Which made me think, oh right, I felt very uncomfortable because I don’t wan’t to be rude but that is totally unnecessary’ (GP trainee 1011; exit interview).

In addition, third-year trainees seemed to rely heavily on their own feelings, checking whether they felt comfortable or had peace of mind after the encounter:

‘I think more of just how did the conversation feel? Did it click? Was I able to express myself well? Did this also get across to the other? Ultimately, it’s about that sense of connection. Did I leave feeling unresolved yet thoughtful, or did I feel satisfied, knowing everything was addressed and understood?’ (GP trainee 1009; start interview).

Third-year trainees emphasized the importance of using their gut feeling in evaluating whether the chosen communication behaviour was effective and if it matched their personal style. When it did, they became more confident in continuing to use that behaviour. They also evaluated their communication behaviour during clinical encounters by observing their supervisors and peers.

#### *Stage 6*–*The trainee internalises the new communication behaviour*

Both the group of first- and third-year trainees experienced a growing sense of ease and fluency in applying new communication behaviours during this stage. They gradually move from a rehearsed, conscious application of these behaviours to a more natural and fluent use, where they experience a sense of mastery or automaticity. Over time, applying these communication behaviours feels less rehearsed, and they are no longer constantly aware of using these behaviours during clinical encounters. Some of the group of first- and third-year trainees reported to experience more mental space to reflect on their communication behaviours, both during and after clinical encounters. For both groups of trainees, internalising new communication behaviours required repeated practice and reflection. Both groups of first- and third-year trainees stressed the importance of focusing on a single aspect of communication for an extended period. Initially, they were very mindful when applying the new communication behaviours. Individual characteristics, such as being curious, enthusiastic, or direct also played a role in the internalisation process, as did personal norms, values and assumptions. The process of internalisation, in all its complexity, was not a straightforward process for any trainee and typically extended over a longer period of time during which they often revisited the first five stages.

First-year trainees particularly stressed the importance of repeated practice to experience a sense of mastery. As one first-year trainee points out:

‘Because in the beginning, it still felt a bit more rehearsed or something. Yes, and perhaps now that’s still the case it still is a bit, but by doing it very often it feels more like something of my own’ (GP trainee 1004; exit interview).

By the time of the exit interviews, first-year trainees noted that scheduling regular follow-up visits with the same patients helped them to experiment with new communication behaviours in a consistent setting. Revisiting earlier learning stages also made them more sensitive to patients’ needs, which enabled them to contextualise their communication behaviour. One first-year trainee expressed this by saying:

‘I have the impression that a lot of sensors are involved, which allow you to feel whether or not something is appropriate or whether you should use this or that. So, then that sensor has become a bit more sensitive. I mean that antenna has grown somewhat, yes, I find it hard to put into words’ (GP trainee 1013; exit interview).

Third-year trainees preferred experimenting with communication behaviours across a variety of clinical situations, such as structuring talkative patients or dealing with medical unexplained symptoms. By the time of the exit interviews, most third-year trainees had no difficulty in identifying the reasons for patients’ visit, though setting boundaries remained a challenge for some. Additionally, third-year trainees who identified as more direct in their approach expressed difficulties with shared decision-making. In such cases, they chose to express their preferences before patients could state theirs. For example, one third-year trainee believed that interrupting the patient too early in history taking might appear rude, which prevented the trainee from to assertively lead the consultation:

‘I always try to treat people very respectfully, so I’m not, I do not tend to interrupt people so soon or I think it’s rude to cut them short, although sometimes that is necessary’ (GP trainee 1002; exit interview).

### Conditions that support learning of skilled communication

We identified conditions that support trainees in their learning across the various learning stages. These conditions were not initiated by any single specific actor but resulted from a dynamic interplay between the trainee, their supervisors and peers, the clinical workplace, and the training institute.

First, it was crucial for first- and third-year trainees to establish a long-term, *constructive relationship with their supervisors*, allowing plenty room for tailored feedback and personalised guidance. Often seen as role models, supervisors therefore played a pivotal role in trainees’ learning. However, in the exit interviews, third-year trainees noted that the relationship with their supervisor was of secondary importance to them as they were more independent and did not see their supervisors on a regular basis compared to first-year trainees. Many first-year trainees indicated that they particularly valued supervisors who, rather than imposing their methods, afforded them the freedom to develop their personal communication styles:

‘She [supervisor] also has more of an exemplary role; she says ‘yes, there’s no need to, you don’t need to become me, right, you are your own doctor’. That is, your own communication style too, so what fits in with it, you know. You should decide that for yourself, choose your own style, right’ (GP trainee 1006; exit interview).

Second, first- and third-year trainees asserted they needed a *nurturing workplace* that afford them both time and space to experiment with new communication behaviours. They particularly valued regularly planned learning activities, such as joint consultation hours. Sharing responsibility for planning these moments with supervisors stimulated continuous development. First- and third-year trainees also emphasized the importance of focusing these learning activities on fostering a safe learning environment rather than on assessment. They indicated the need of more time during the clinical encounters. Indeed, having more time made first-year trainees feel more comfortable and gave them more opportunities to experiment with new communication behaviours, whereas third-year trainees wanted more time to support their personal growth or to prepare educational assignments. Furthermore, first- and third-year trainees stressed the importance of a workplace that facilitates ample exposure to challenging patient cases aligned with their learning goals. This would give them more opportunities for repeated practice, in turn boosting their confidence in applying the new communication behaviour.

In addition, both groups of trainees identified that *communication training needed to be aligned with clinical practice*. According to third-year trainees, the standardised consultation structures provided during training sometimes failed to capture the authenticity and complexity of clinical encounters:

‘I also think that the most effective consultations are often the consultations in which you actually do not follow that MAAS global. So, it’s better to deviate from that. For various reasons. For instance, it’s often difficult to evoke those emotional reflections and empathy somewhere in a standardised consultation. They must come from the moment itself and then that MAAS global is like a straitjacket preventing you from giving that emotional reflection and empathy at the right time (GP trainee 1011; start interview).

First-year trainees echoed these concerns, although they did appreciate the guidance these structures offered in moments of uncertainty about how to proceed in the clinical encounter. First- and third year trainees also suggested that communication training should concentrate on a single aspect of communication to help them narrow down their focus of attention in the encounters. These communication behaviours then needed to be discussed regularly in the training sessions and teachers should give continuous feedback to foster trainees’ development. Third-year trainees specially valued in-depth discussions with peers during training sessions, which should ideally take place in small groups to foster a safe atmosphere for sharing experiences. Both group of trainees agreed that trainings offered by their institutes were supportive of their learning and highlighted that it should also allow them the opportunity to develop their own communication style. A few first-year trainees noted that collaborative group reflection offered insights into how their belief system influenced recurring communication patterns, thereby contributing to the development of new communication behaviours. The following third-year trainee also explained that trainees’ teachers could promote their development:

‘Because that [teacher] also pays attention to that deeper level of why are you doing that and of course he doesn’t look at your direct patient contact, but rather at your reasoning behind it, why you are doing certain things. (GP trainee 1004; start interview).

Another condition that emerged was to support trainees’ learning was *assessments with narrative feedback*. First- and third-year trainees pointed out that feedback was perceived most useful when it was presented in a narrative form. Consequently, it was easier and more meaningful for them to interpret than when feedback was given numerical. During communication assessments, they valued the dialogue that was offered by the institute with the assessor to discuss the provided feedback.

The fifth condition that supported trainees’ learning included having dedicated *time for mandatory reflections*. First-and third-year trainees reported receiving regular feedback on the job by their supervisors or peers through informal assessments with their supervisors. However, they were not used to document these feedback and reflection moments in their portfolios. As a result, they felt that the mandatory reflections required by their training institutes were redundant, because they had already reflected on feedback in learning conversations with their supervisors. Since no time was set aside for these mandatory reflections during educational days or in clinical practice, it felt like just an extra task that added to their workload. However, they did acknowledge the importance of purposefully allocating time for these mandatory reflections, as it could enhance their awareness of communication behaviours and clarify their learning goals.

## Discussion

In this study, we aimed to deepen our understanding of how GP trainees develop skilled communication during the first and third years of the training programme, focusing on the longitudinal development of communication performance during workplace learning We constructed a six-stage conceptual model that incorporated the used learning strategies of both first-and third-year trainees, as well as the conditions that supported their learning. Learning started with an impactful experience that raised trainees’ awareness of their communication behaviour, prompting them to look for alternative communication behaviours. After experimenting, trainees evaluated the effectiveness of these new communication behaviours. It was through repeated practice, that these behaviours became internalised. The conditions that supported trainees’ learning were: a constructive relationship with their supervisors, a nurturing workplace, alignment between training and practice, narrative feedback from formative assessments, and time for mandatory reflections.

Our conceptual model aligns with Kolb’s experiential learning model (Kolb, 1984), commonly used in workplace learning. Kolb defined learning as ‘*the process whereby knowledge is created through the transformation of experience’.* This experiential learning model outlines a learning cycle consisting of four stages: encounter a concrete experience, reflecting on the experience, reflective observation, abstract conceptualization, and trying out new behaviours through active experimentation. We offered additional insights by demonstrating that the identified learning strategies in our conceptual model seemed to evolve over time. Likewise, our model bears a resemblance to existing conceptual models that visualise trainees’ communication learning process (Giroldi et al., 2017; van den Eertwegh et al., 2015). We aimed to enrich these existing models by providing an in-depth exploration of how learners develop skilled communication and what supports this process as they navigate through the identified six stages of our conceptual model. Our conceptual model supports the content validation of the existing conceptual models (Giroldi et al., 2017; van den Eertwegh et al., 2015), highlighting the importance of reflection to become aware of communication behaviours, as well as repeated practice and the evaluation of the effectiveness of behaviours to internalise these communication behaviours into trainees’ repertoires.

In the conceptual learning models of van den Eertwegh et al. and Giroldi et al., the authors have theorised that impactful experiences trigger learning (van den Eertwegh et al., 2015; Giroldi et al., 2017). We found that triggers, such as emotions or challenges faced in clinical encounters, varied among trainees. Yet, as Eraut notes learners might have difficulty in recognising these impactful experiences as learning opportunities (Eraut, 2004). Additional mediators were trainees’ sensitivity to these triggers or their willingness to learn from them (Sehlbach et al., 2020). We saw that reflection helped render these impactful experiences more transparent and explicit (Epstein et al., 2008; Eraut, 2004; Johnson et al., 2023).

Subsequently, trainees became aware of their communication behaviours and deepened their understanding through different reflective practices, such as discussing video-taped consultations with peers and using communication interaction models during communication training. As Ng and colleagues explain, reflection is not a fixed skill, but a dynamic process that develops from personal experience and various sources of knowledge (Ng et al., 2020). As proposed by Schön (Schön, 1991), distinguishing between reflection*-in*-action and reflection-*on*-action is important because communication is a fast, on-going process that leaves little room for reflection without disrupting the natural flow of the consultation. In our study, reflection-*in*-action happened implicitly during the encounter, whereas reflection*-on-*action took place after the clinic, providing trainees time to think and purposefully reflect on their communication. However, the extent of trainees’ engagement in this reflection varied: whilst some trainees used questions to stimulate reflection, others relied on external support from supervisors. Similarly, trainees employed various methods to monitor their communication behaviour. By reviewing videotaped-consultations (self-observation), for instance, trainees were able to recognise inappropriate postures such as crossed arms (self-judgement) and adapted their behaviour accordingly by reminding themselves to adopt an open posture (self-reaction) (Epstein et al., 2008; Johnson et al., 2023). The reflective and self-directed nature of workplace learning in medical education is a crucial element in the professional development of both trainees and doctors, although this is not always adequately supported in practice (van de Wiel et al., 2011).

By providing tailored feedback and guidance, supervisors served trainees with concrete examples of new sentences. In experimenting with the new communication behaviours across contexts, trainees did not only imitate the behaviours learnt from supervisors, but also adapted and refined them to be in line with their communication styles (Billett, 2014; Wulf, 2008). To facilitate the transfer to clinical encounters, trainees first evaluated these behaviours, which proved challenging in several ways. Evaluating communication behaviour often involves self-assessment, yielding reflections that become generalized (Johnson et al., 2023). Unlike self-monitoring, a general evaluation lacks specificity of context and time as it is not necessarily related to one specific situation, nor does it immediately follow a clinical encounter (Johnson et al., 2023). The evaluation process of communication behaviour may also be challenging due to the absence of guidelines or assessment criteria (Salmon & Young, 2011; Verheijden et al., 2023a). Becoming a skilled communicator is a personalised process of recognising attitudes, feelings and thoughts (Salmon & Young, 2011; Zoppi & Epstein, 2002).

When it comes to internalising communication behaviours, this was perceived as a sense of automaticity and of fluency in applying communication. This finding is consistent with a recent study by Kawamura and colleagues, who distinguished between conceptual understanding and procedural fluency in developing communication expertise (Giroldi et al., 2017; Kawamura et al., 2020; Sehlbach et al., 2020). Using humour to comfort patients, for instance, demonstrates procedural fluency, whereas trainees becoming more sensitive to patients’ needs, enabling them to contextualize their communication behaviours through repeated practice, reflects conceptual understanding (Carrard et al., 2018; Kawamura et al., 2020).

Our findings support that workplace learning is implicit, as trainees learn by doing (Eraut, 2004). More specifically, trainees learned from informal assessments with supervisors during clinical encounters. Learning was supported by collaborative group reflection with peers and listening to communication-focused podcasts. In line with Sagasser et al., we identified that trainees’ communication behaviours were shaped by engaging in short and long loops of informal learning (Eraut, 2004; Sagasser et al., 2012). Loops were short when changes in behaviours were minor (e.g., switching to simpler language when a patient looked confused) and longer for more complex skills (e.g., interviewing patients with psychological complaints) (Sagasser et al., 2012). To internalise communication behaviour, it was essential that trainees continuously and repeatedly re-entered both types of learning loops. Consequently, trainees adapted communication behaviour not only *during* the encounter, but also *across* encounters (Sagasser et al., 2012). It follows that internalising behaviours permeated our conceptual model throughout whereas experimenting with new communication behaviours, was frequently revisited. The fact that there was no universal pathway identified in our study to attain expertise in communication underscores the complexity of clinical practice.

Regarding conditions that supported learning, trainees valued supervisors who, rather than imposing their own methods, acted as coaches and allowed them to develop their unique communication repertoires. Such a personalised approach requires an educational dialogue in which trainees and supervisors share knowledge to establish learning goals (Muhonen et al., 2021). A nurturing workplace also proved vital in trainees’ learning to develop expertise, especially one that, consistent with deliberate practice theory offered regular opportunities to practise and evaluate communication behaviour (Hoffman & Donaldson, 2004; Swanwick, 2005; Ericsson, 2006; Zimmerman et al., 2006; van de Wiel et al., 2011). Ericsson argues that developing expertise requires deliberate practice, including actively setting new learning goals to counteract automatic behaviours (Ericsson, 2006, 2008). Billett builds further upon Ericsson’s line of argument by emphasising the interdependence between learning and the workplace, highlighting the importance of affording learning activities, such as joint consultation hours, tailored to trainees’ learning preferences to stimulate reflection, evaluation, and repeated practice (Billett, 2001, 2004, 2016). Yet, workload and time constraints often hindered reflection and learning (van de Wiel et al., 2011; Hoffman & Donaldson, 2004), highlighting the need for workplace climates that facilitate learning activities (Billett, 2001).

Trainees also emphasized the need of alignment between communication training and actual clinical practice. Training should adopt a learner-centred approach, in balancing learning activities with routine practice and reflective activities (de la Croix & Veen, 2018; Schaepkens et al., 2023). Unsurprisingly, narrative feedback from formative assessments was perceived by trainees as valuable to support their learning. Watling and colleagues emphasised that well-timed and tailored feedback was essential for trainees to support their learning (Watling et al., 2012).

### Strengths and limitations

Our findings should be interpreted with a few strengths and limitations in mind. In terms of strengths, we used a novel approach to explore how trainees learn communication in the workplace. We adopted a longitudinal design and triangulated data sources using stimulated recall interviews, observations and audio diaries. By doing so, we minimised the risk of data loss that may occur in longitudinal qualitative research when data are treated as separate units that remain static over time (Balmer et al., 2021; Smithbattle et al., 2018). Our use of audio diaries reduced recall bias, as its recording activity encouraged participants to continuously reflect on their learning experiences. The use of a mobile application for these audio diaries might also have created a safe space for participants to share their experiences, which is critical when narrating them (Clandinin et al., 2017). The risk of such bias was further reduced by the fact that the interviews immediately followed the clinical observations. Most importantly, the study encouraged deep reflection on trainees’ tacit communication experiences and associated behaviours (van Braak et al., 2018). The interviews helped render trainees’ learning process more explicit. Finally, this study took place at two out of the eight GP training institutes in the Netherlands, with different educational cultures potentially enhancing the generalisability of our findings.

As for the study’s limitations, we should mention our small sample size of 13 participants. Yet, considering the intensive data collection methods used, this sample size proved sufficient to obtain a rich dataset that could address our research question (Atherley et al., 2021; Malterud et al., 2016). Next, the researcher-participant relationship, as discussed in the Reflexivity paragraph, presents a dual aspect, serving both as a strength and a limitation. To minimise researcher bias, we employed strategies such as regular discussions of findings within the research team and conducting clinical observations in pairs.

The third limitation lies in the use of purposive sampling, which potentially limits the generalisability of findings. This approach may have biased our sample towards trainees participating that are more inclined to enhance their communication learning. Nevertheless, we still deem our findings relevant to the GP realm because our aforementioned data triangulation yielded a comprehensive dataset, offering a more in-depth and thick description of trainees’ workplace learning (Frambach et al., 2013) which adds to and validates the previously identified conceptual learning models.

An important consideration is the reflective interventions introduced through the use of stimulated-recall interviews and audio diaries, in which trainees were instructed to reflect continuously on their communication learning experiences. Most trainees experienced these interventions as mindfully challenging, as they were encouraged to deliberately take time for reflection, enhancing awareness of their communication learning process and the strategies used as well as promoting future learning. Whilst these interventions encouraged reflection on communication behaviour, this may not provide insight into educational reality. As the interventions may have resulted in a more explicit and idealised perception of trainees’ learning process. This reservation was not explicitly accounted for in our conceptual model which presented various pathways to learning.

### Implications

Our findings may have implications for both future research and training. An interesting pathway for research would be to explore if similar learning processes exist in other medical contexts. This would support the transferability of our findings.

As for training, the use of planned reflective assignments could be a promising approach to support learners in reflecting on their communication performance, consequently leading to raised awareness and triggering learning. To connect practical implications with each of the six stages identified in the conceptual model, we propose: (1) *Triggering stimuli:* learning activities, such as discussing trainees’ video-taped consultations during learning conversations, offering clinical opportunities to practice with shared impactful experiences – like structuring consultations for patients with psychological symptoms versus somatic symptoms — and performing joint consultation hours and individual consultation hours with exposure to challenging patient cases aligned with trainees’ learning goals, could trigger learning; (2) *Raising awareness:* supervisors highlighting specific communication behaviours to reinforce trainees’ recognition of this behaviour during observations. Furthermore, training may help raise trainees’ awareness of communication behaviour by allocating time for mandatory reflections following formative assessments. These reflections could be followed by educational dialogues aimed at deriving meaning from the reflection process, fostering deeper understanding and insight into trainees’ learning goals; (3) *Identifying:* formal raining can assist trainees in discovering alternative communication behaviours by providing diverse resources such as skills training, role-plays, and facilitating peer learning through discussions on impactful experiences from their consultations and video-taped consultations. Additionally, workplaces can enhance this process by offering opportunities for trainees to observe their supervisors or GP colleagues; (4) *Experimenting:* various clinical experiences in both the GP clinic and other contexts (i.e., house calls, GP emergency services) can inspire trainees to experiment with new communication behaviours, helping them develop a unique communication repertoire. Furthermore, supervisors can support trainees by offering concrete sentence examples to experiment with; (5) *Evaluating*: workplaces could help trainees incorporating patient feedback and facilitating discussions with supervisors through the regular planning of learning activities in a safe environment where trainees feel free to seek feedback, experiment and evaluate communication behaviours. Additionally, GP teachers and peers can further enhance trainees’ evaluation process by fostering self-monitoring of communication behaviour during formal training. For example, this can be achieved through joint review sessions of trainees’ video-taped consultations, providing time for constructive criticism of applied communication behaviour; (6) *Internalising*: by integrating repeated practice with opportunities for trainees to revisit previous stages, along with tailored feedback and reflection, may enhance longitudinal monitoring of learning progress. For instance, workplaces could support trainees by scheduling consultations with similarly impactful patient cases that trigger learning and by installing regular learning activities, such as reflective assignments and discussions, to stimulate the development of their personal communication repertoire Additionally, it is crucial to acknowledge that trainees have different learning needs and approaches. There is no one-size-fits-all solution in terms of recommended learning strategies for communication (Sehlbach et al., 2020).

## Conclusion

Our study revealed a six-stage cyclic conceptual model including learning strategies and supportive conditions to develop skilled communication during workplace learning. Developing skilled communication requires a continuous, circular approach, emphasizing repeated practice, reflection, and tailored feedback to help learners self-monitor their communication behaviours and adapt these behaviours flexibly during clinical encounters. To facilitate such an approach, we recommend that trainees are supported with reflective learning activities to stimulate the development of their personal communication repertoires. For clinical workplace learning, it is essential to create a safe learning environment with regular planned learning activities guided by supervisors who serve as coaches. Furthermore, we suggest that aligning communication training with clinical workplace learning is essential for helping trainees to develop and internalise their communication behaviours through a balance of combining repeated practice and reflective learning activities.

## Supplementary Information

Below is the link to the electronic supplementary material.

Supplementary file1 (DOCX 22 KB)

Supplementary file2 (DOCX 22 KB)

Supplementary file3 (DOCX 275 KB)

Supplementary file4 (DOCX 19 KB)

Supplementary file5 (DOCX 15 KB)

Supplementary file6 (DOCX 16 KB)

## Data Availability

No datasets were generated or analysed during the current study.
