# Supplementary material for: Unravelling the art of developing skilled communication: a longitudinal qualitative research study in general practice training
Source: Adv Health Sci Educ Theory Pract. 2024 Dec 17;30(4):1231–55. doi: 10.1007/s10459-024-10403-6 (PMC12391227; doi:10.1007/s10459-024-10403-6)
Supplement: Supplementary file 1 — Supplementary file1 (DOCX 22 KB) [file 10459_2024_10403_MOESM1_ESM.docx]

Supplementary information - Appendix A – Interview guide start-interview and clinical observation

Article title: Unravelling the art of developing skilled communication: a longitudinal qualitative research study in General Practice training

Journal name: Advances in Health Sciences Education - Theory and Practice

Author names; Michelle Verheijden^1,2^; Angelique Timmerman1, Dorien de Buck, Anique de Bruin^2^, Valerie van den Eertwegh^2^, Sandra van Dulmen^3^, Geurt Essers, Cees van der Vleuten^2^, Esther Giroldi^1,2^.

Affiliation:

1. Care and Public Health Research Institute (CAPHRI)
2. School of Health Professions Education (SHE)
3. Netherlands Institute for Health Services Research, Utrecht, Netherlands (NIVEL)

E-mail address of corresponding author: [m.verheijden@maastrichtuniversity.nl](mailto:m.verheijden@maastrichtuniversity.nl)

**In preparation of the interview** – materials needed:

- Informed consent forms signed by both trainee and supervisor
- Patient information letters and printed patient informed consent forms
- Field notes forms (digital or printed)
- Audio recording equipment
- Patient list for the clinic printed by trainee
- Demographic data form printed for trainee to fill in
- Pen, paper, and laptop

**Introduction for start of observation patient encounter** – Introduce yourself and let the participant introduce themselves

- Thank you for your time today and your willingness to participate in this research.
- At times, I will read the interview guide verbatim to ensure consistency among all participating trainees and minimize variation.
- Before we begin the observation of your clinic, I will explain the practical details and the purpose of the research.
- Voluntary participation. Ensuring privacy: "Firstly, your participation in the research is voluntary, and you may choose to withdraw at any time without giving a reason. Privacy is guaranteed by using pseudonyms for the collected research data." This means that the data we collect now (audio recordings and notes) will be assigned a code that cannot be directly linked to you or individual patients but only indirectly through another securely stored file.
- Explaining the research purpose: "I would like to tell you something about the research's purpose. As mentioned in the information letter, this study focuses on how trainees develop skilled doctor-patient communication during general practitioner training. Skilled communication includes all communication between the doctor and the patient. While the literature has extensively described how trainees do this in theory, there has been little research into how this learning occurs in daily practice and how the training can best support trainees." Is the research purpose clear, or do you have any questions?
- Explaining the research procedure: the research today consists of two parts. First, we will observe your clinic, and after a short break of 30-60 minutes, we will conduct the interview, which will last a maximum of one hour. It would be most helpful if you have a list of the patients you saw during your clinic for reference during the interview.
- If you have no further questions, I would like to suggest starting your clinic and our observation of it. We prefer to sit behind the patient and will not speak during the consultation itself. Please conduct your consultations as you would if we were not present. We will take notes and, with your and the patient's consent, record the conversation using an audio recorder.
- Requesting permission for audio recording: "I would like to request your permission, if it's acceptable to the patient as well, to make an (audio) recording during each consultation in your clinic. Is that ok for you if I record your encounters when patients have given consent?
- Do you have any questions at this moment

**Clinic Observations -** Based on field notes and audio recordings

- Take a seat in the consultation room, preferably behind the patient, and briefly introduce yourself to the patient by mentioning your name after obtaining consent for observation.

**Introduction to stimulated-recall Interview**

We have just observed your clinic, and now I would like to transition to the interview. I would like to explain how we will proceed and the purpose of the interview.

- Purpose and procedure of the interview: "The goal is to review your clinic using observations of your consultations to map your learning moments." The interview will last a maximum of one hour.
- Requesting permission for audio recording: "I would also like to request your permission for making an (audio) recording during the interview. Is that ok for you if I record this interview? I am now going to start the recording [start recording]

**1. Level of concrete communication behaviour (experiences) - cue: valuable experiences regarding communication behaviour of trainee**

- I would like to ask you to open your agenda and the patient list from your clinic today. First, I would like to ask you:

| Starting with valuable experience of trainee: "As you look at this list, what was a valuable communication experience for you during the clinic?"   - What makes you describe this as a valuable experience? - Can you describe this experience from your perspective as a trainee? |
| --- |
| Reflection on valuable experience: ‘Can you think aloud about what was going through your mind during the consultation?’   - How did you experience the situation and the communication behaviour? What were your thoughts and feelings? - What was your goal in using this communication behaviour? Did you have any other goals? - Can you empathize with how the patient experienced it and their reactions? |
| Influencing factors   - Can you think back and consider which factors (from yourself/patient/practice) influenced your communication behaviour? |
| Alternative communication strategies: ‘Thinking back, could you have used your communication differently in this consultation?’   - What other choices did you have? Can you describe the reasons for choosing to use or not use this communication in this consultation? |
| Connecting specific valuable experience to learning   - Thinking back, are you satisfied with your applied communication in this consultation? - What insight does the discussion of this experience provide you with now? What will you take away from our conversation? - Do you intend to do things differently in a similar situation, and can you explain this? - Have you experienced the effectiveness of this applied communication before, and if so, in what situations?   - Can you share something about that?   - How did you learn to apply it? What helped you learn this communication? |

**2. Meta-level concrete communication behaviour experiences – focus on reflecting on own learning processes in daily practice with the help of a cue (audio recording of clinic, stimulated recall)**

- We have just extensively discussed one (or more) valuable communication experiences for you. Now, I would like to take a broader look at learning itself: how you learn communication in your daily practice. To understand this better, I would like to ask you to choose a consultation from your list that you think might be relevant to your own communication learning goals.
  - (If the AIOS finds it difficult to choose a consultation, provide guidance based on the observations made to stimulate recall)
  - (Coordinate with the AIOS which fragment will be reviewed and why the choice falls on that fragment. Explore their considerations and which fragment they would like to review); Why is it important for you to choose this consultation?
- Once you've chosen a consultation, I will retrieve the audio recording and instruct you to stop the recording at the moment that is important for your learning about communication. Can you give me a direction within the consultation where this moment occurred? If not, I will play the recording from the beginning.
- Listen to the audio recording of the consultation
  - Note the consultation and the minute number.
- Now that we have listened to the moment you identified as potentially relevant to your communication learning goals, I would like to ask you:

| Understanding the trainees learning strategies   - Why is this recording relevant to your learning about communication now?   - How do you choose it? - Would you normally formulate a learning goal based on this recording?   - How do you go about it? What's your process?   - What do you base this choice on? Where does it come from?     - Are your learning goals specific or more general? - Would you work on this learning goal?   - Why or why not? Can you explain how? - How are you typically motivated to learn about communication?   - What lies behind this motivation? Why does it motivate you? |
| --- |
| Transitioning to a meta-level of learning   - How do you typically assess where you stand in doctor-patient communication (when discussing the overarching theme in your training)?   - What do you consider important in that assessment?   - Do you ask specific or more general feedback questions? What guides you in this? - How do you envision yourself as a proficient communicator, and how far along are you in your own development? - When do you learn doctor-patient communication? Under what conditions? And can you provide an example?   - Examples: discussions with your supervisor, return sessions, assessments, what you've observed yourself - How do you evaluate the effectiveness of learning communication?   - When are you personally satisfied with the outcome?   - When do you consider your learning goals achieved? - Could you provide a concrete example or something you are satisfied (or dissatisfied) with in terms of communication? |
| Conditions supporting/inhibiting learning   - What do you need to be supported in your learning, both from the training program and from the practice?   - What would that look like?   - Can you explain why it would help you?     - Examples: Would you normally have recorded the consultation? Or will you bring it up during a return session or review it again yourself? Or will you immediately practice different behavior? Or will you discuss it with your supervisor? - Would you discuss this experience with others (e.g., fellow AIOS, supervisor, mentor, or colleague GP)? Why or why not? And with whom? |
| Reflection on interview: ‘I have intentionally asked you about your learning experience. What insight does our discussion provide you with now? What will you take away from our conversation?’ |

**3. Discuss how formal communication training sessions at the training institute impacts learning**

- How do you perceive the emphasis on training doctor-patient communication during training sessions at the training institute?
- How do you currently experience the doctor-patient communication training sessions and its alignment with clinical practice?
- How do you currently experience the assessment of doctor-patient communication and its alignment with clinical practice? (with a focus on the impact of assessment on learning communication)

**4. Closing questions and explaining procedure of audio-diary and follow-up interview (exit-interview)**
