# Supplementary material for: Unravelling the art of developing skilled communication: a longitudinal qualitative research study in general practice training
Source: Adv Health Sci Educ Theory Pract. 2024 Dec 17;30(4):1231–55. doi: 10.1007/s10459-024-10403-6 (PMC12391227; doi:10.1007/s10459-024-10403-6)
Supplement: Supplementary file 2 — Supplementary file2 (DOCX 22 KB) [file 10459_2024_10403_MOESM2_ESM.docx]

Supplementary information - Appendix B – Interview guide exit-interview and clinical observation

Article title: Unravelling the art of developing skilled communication: a longitudinal qualitative research study in General Practice training

Journal name: Advances in Health Sciences Education - Theory and Practice

Author names; Michelle Verheijden^1,2^; Angelique Timmerman1, Dorien de Buck, Anique de Bruin^2^, Valerie van den Eertwegh^2^, Sandra van Dulmen^3^, Geurt Essers, Cees van der Vleuten^2^, Esther Giroldi^1,2^.

Affiliation:

1. Care and Public Health Research Institute (CAPHRI)
2. School of Health Professions Education (SHE)
3. Netherlands Institute for Health Services Research, Utrecht, Netherlands (NIVEL)

E-mail address of corresponding author: [m.verheijden@maastrichtuniversity.nl](mailto:m.verheijden@maastrichtuniversity.nl)

**In preparation of the interview** – materials needed:

- Patient information letters and printed patient informed consent forms
- Field notes forms (digital or printed)
- Audio recording equipment
- Patient list for the clinic printed by trainee
- Narrative containing a summary of start-interview including observation and audio-diaries
- Pen, paper, and laptop

**Introduction for start of observation patient encounter**

- Thank you for your time today and your willingness to participate in this research.
- At times, I will read the interview guide verbatim to ensure consistency among all participating trainees and minimize variation.
- We have met six months ago (introduce again if necessary). Before we begin the observation of your clinic, I will explain the practical details and the purpose today.
- Voluntary participation. Ensuring privacy: "Firstly, your participation in the research is voluntary, and you may choose to withdraw at any time without giving a reason. Privacy is guaranteed by using pseudonyms for the collected research data." This means that the data we collect now (audio recordings and notes) will be assigned a code that cannot be directly linked to you or individual patients but only indirectly through another securely stored file.
- Explaining the purpose of today: "Do you still have a clear understanding of the research goal, or would you like me to explain it? Our focus is on how AIOS learn during their general practitioner training when it comes to proficient doctor-patient communication. By this, I mean all communication that takes place between the doctor and the patient. The goal for today is to map your learning over the past six months of your training, using information from the start-interview and the learning moments you have recorded in your audio diary.
- Explaining the research procedure: As you may already know, the research today consists of two parts again. We will first observe your clinic, and after a short break of 45-60 minutes, we will proceed with the interview, which will last a maximum of one hour. If you have no further questions, I would like to propose that we begin with your clinic and the observations of your consultations. We prefer to sit behind the patient again and will not talk during the consultation itself. Please conduct your consultations as you would if we were not present. We will take notes of the consultations, and if both you and the patient agree, we will record the conversation using an audio recorder."
- If you have no further questions, I would like to suggest starting your clinic and our observation of it.
- Requesting permission for audio recording: "I would like to request your permission, if it's acceptable to the patient as well, to make an (audio) recording during each consultation in your clinic. Is that ok for you if I record your encounters when patients have given consent?
- Do you have any questions at this moment?

**Clinic Observations -** Based on field notes and audio recordings

- Take a seat in the consultation room, preferably behind the patient, and briefly introduce yourself to the patient by mentioning your name after obtaining consent for observation.
- The narrative of the AIOS, based on the earlier observation, interview 1, and the audio diary, guides the observation of the AIOS's clinic and documents any notable observations during the clinic.

*During the break between observation of the patient encounter and the exit-interview, the two researchers discuss the observation in relation to the trainees narrative to identify valuable moments that can be revisited in the observed clinical encounter. These moments are then incorporated into the interview if the trainees has difficulty selecting a valuable learning experience*.

**Introduction to stimulated-recall interview**

We have just observed your clinic, and now I would like to transition to the interview. I would like to explain how we will proceed and the purpose of the interview.

- Purpose and procedure of the interview: The aim is to reflect on your learning in doctor-patient communication over the past six months, using one of your consultations from today's clinic. We have a brief summary of the earlier observation of your clinic, the start interview, and your recorded learning moments as tools to discuss which themes have been important in your learning and to map them out.
- Requesting permission for audio recording: "I would also like to request your permission for making an (audio) recording during the interview. Is that ok for you if I record this interview? I am now going to start the recording [start recording]

**1. Review of trainees’ learning/learning process over the past six months**

- I would like to ask you to open your agenda and the patient list from your clinic today. First, I would like to ask you:

| Starting with valuable learning experience of trainee (here and now): ‘When you look at this list, what was a valuable experience for your learning in communication during your clinic?’   - What is the reason for you to describe this as a valuable experience? - What made this a valuable experience for you?   - Elaborate by asking, for example: How does the valuable learning experience relate to personal learning in communication? |
| --- |
| Place valuable learning experience in the context of learning over the past six months and the first interview (back, process):   - Have you seen this/these theme(s) reoccur in your learning over the past six months? If yes, how can you explain it? - Does this theme align with your development in learning doctor-patient communication? If yes, how does it align, and how has your development been? Where do you stand in it? - Can you describe what your learning has looked like concerning this theme? - Would you discuss these learning experiences as examples in your education or with your supervisor? What feedback have you received about them? - What has helped you in learning about these themes? How have you tried to implement this in practice? Who or what has helped you? Would you discuss these learning experiences as examples in your education or with your supervisor? What feedback have you received about them? - What has potentially hindered your learning about these themes? - Have these themes changed? How?   - Pay attention to probing: How has (theme) become a part of your learning? What helps you become aware of where you stand in learning this? |
| In the first interview, we discussed how you learn doctor-patient communication in practice and what helps you. Do you feel that this has changed over the past six months? Do you feel that certain things have become less or more important? How have you observed this? (back, process) When you look back at the first interview six months ago, do you recognize themes/these themes you've been working on in your learning over the past six months? And how? |
| Placing the valuable learning experience in the future (forward, process): Thinking back to today's clinic that we just discussed, do you think you could have used your communication differently in this consultation (here and now)? o How will you work on this in the upcoming period? What does this mean for your learning in the near future? (forward) o What do you need in the future? (forward, process) |
| - Has anything changed in how you view learning doctor-patient communication in the past six months (back, process)? Can you tell me more about that? - Have any other themes emerged during your clinic today that have been important for your learning of doctor-patient communication? If yes, what are these? (here and now) o If the AIOS mentions a theme, inquire about it in the same way as outlined above, starting with placing the theme in the learning of the past six months. Or, if the researcher perceives missing themes from the narrative, make them discussable here. Mention the theme and ask the AIOS how it relates to their learning. |

**2. Transition to audio-diary**

- I would like to transition to the audio diary and the role it played in your learning over the past six months (back, audio diary)

| Evaluate technical aspect: ‘How did you experience recording your learning experiences?’   - Did you encounter any issues with recording or speaking about your learning experiences? - Did you come across anything unexpected? |
| --- |
| How did the recorded learning experiences contribute to your learning of doctor-patient communication over the past 6 months (back, process)?   - Escape: If the AIOS mentions that it hasn't contributed to learning doctor-patient communication, focus on themes identified in the narrative, such as those related to learning strategies. |
| Did the audio diaries lead to the emergence of new themes?   - How did you work on these themes in your learning over the past months? |
| Looking back at your own learning and what you've been working on, how does this align with the learning experiences you recorded in your audio diary?   - If the AIOS finds it challenging to think about this, provide guidance based on the narrative, or if the AIOS doesn't mention a theme that's important from the narrative, make it discussable here. For example, when thinking back to your audio diary, you mentioned (a theme identified by the researcher). Have you been working on that in your learning? Why or why not? - If necessary, ask the AIOS for more clarification on what they mean by the recorded learning experience. - Can you think out loud about why this was valuable for you? Or, why it wasn't valuable for you? |
| Placing the learning experience from the audio diary in the clinic itself (here and now)   - Is this theme/learning experience now relevant for your learning in doctor-patient communication when you think about your clinic just now and the consultation we discussed?   - Why or why not?   - What has helped you in learning? What has hindered you? |
| Placing the learning experience from the audio diary in the future (here and now)   - If you now look into the future, what does this learning experience/theme mean for your learning in the coming period? What will you take away from this for yourself? - What do you need in the future? (forward, process) |

**4. Connecting learning communication to becoming a skilled communicator (forward, future)**

- During our first interview, we discussed how you viewed a skilled communicator and how far you were in developing into one.
- Can you still articulate how you saw a skilled communicator back then? If the trainee can't recall, help them answer the question by mentioning how the trainee viewed a skilled communicator.
  - Has anything changed compared to six months ago (back, process)?
  - When you look into the future now, what do you take away from this for yourself? What would you still like to learn? (forward)
  - How do you envision yourself as a skilled communicator in the future?

**5. Evaluate interview:** How did you experience the interview and reflecting on your learning moments related to doctor-patient communication?

**6. End interview and thank participant**
