# Supplementary material for: Unravelling the art of developing skilled communication: a longitudinal qualitative research study in general practice training
Source: Adv Health Sci Educ Theory Pract. 2024 Dec 17;30(4):1231–55. doi: 10.1007/s10459-024-10403-6 (PMC12391227; doi:10.1007/s10459-024-10403-6)
Supplement: Supplementary file 3 — Supplementary file3 (DOCX 275 KB) [file 10459_2024_10403_MOESM3_ESM.docx]

Supplementary information - Appendix C – Protocol how to use the smartphone application ‘Siilo’

Article title: Unravelling the art of developing skilled communication: a longitudinal qualitative research study in General Practice training

Journal name: Advances in Health Sciences Education - Theory and Practice

Author names; Michelle Verheijden^1,2^; Angelique Timmerman1, Dorien de Buck, Anique de Bruin^2^, Valerie van den Eertwegh^2^, Sandra van Dulmen^3^, Geurt Essers, Cees van der Vleuten^2^, Esther Giroldi^1,2^.

Affiliation:

1. Care and Public Health Research Institute (CAPHRI)
2. School of Health Professions Education (SHE)
3. Netherlands Institute for Health Services Research, Utrecht, Netherlands (NIVEL)

E-mail address of corresponding author: [m.verheijden@maastrichtuniversity.nl](mailto:m.verheijden@maastrichtuniversity.nl)


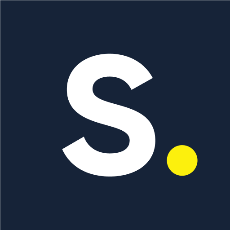
Participants were provided with information about the smartphone application ‘Siilo’ and received a protocol how to use the application for making an audio-diary and how to download it. In the following paragraphs, we have outlined the aforementioned steps in the same way as the participants received it.

**General information about the smartphone-application ‘Siilo’**

*Siilo* is an application specifically designed for medical professions and respects the highest compliance standards regarding privacy; all data is end-to-end encrypted, users are authenticated and all data is deleted after 30 days. Within *Siilo* a group chat will be created for each trainee separately with the first researcher (MV) and a research assistant to share audio recordings safely and to send reminders for capturing learning moments.

**How to use the smartphone-application ‘Siilo’**

We kindly request you (participant) to maintain an audio diary for a duration of 6 months, where you audio-record a learning moment (up to 10 minutes) every two weeks. Specifically, this means we are asking you to audio-record 12 learning moments related to your communication behaviour as a trainee, in which doctor-patient communication is central. The purpose of the audio diary is for you to share at least one learning moment related to your communication (with patients). Feel free to share whatever you had like, regardless of timing, and what you have learned.

Learning moments are those that have been informative for you or have led to insights. These moments can spontaneously arise from your own patient interactions, such as a significant patient encounter where you realized the consultation did not go well or a consultation in which you were satisfied with your communication behaviour. However, learning moments can also occur at other moments in time, such as during a discussion with your supervisor or during educational sessions at the training institute. Furthermore, learning moments can result from self-awareness and insight into your own communication, like employing a dual-track approach for patients with medically unexplained symptoms. At the end of the day or in the evening, when you may have more time to reflect on your communication behaviour and what happened during the day: "Did it go as I intended? Is there something that stuck with me today? Could I have done it differently?" You can use these questions as a guide when recording a learning moment. Try to describe your learning moment and reflect on it.

For recording and securely sharing your learning moments, we use the Siilo application. *Siilo* is an application specifically designed for medical professions and respects the highest compliance standards regarding privacy. Once you have sent us the consent form for study participation, we will send you a Siilo invitation to connect via the Siilo app. Within *Siilo* a group chat will be created for each trainee separately with the principal researcher (Michelle Verheijden) and a research assistant (Paddy Hinssen) to share audio recordings safely and to send reminders for capturing learning moments. The group chat is only accessible to the principal investigator and the research assistant. Please refer to the 'How to download the Siilo App' guide for more information on downloading the application.

The recorded learning moments will be temporarily stored in the chat (up to 30 days), and shared learning moments will be securely saved on a hard drive every two weeks. After storage, the principal investigator or research assistant will remove the shared learning moments from the chat. We kindly request you to use your phone's dictation function to record the learning moments and then send them via the chat function of the Siilo app. Once the recording is complete, locate the 'recorded learning moment' in your phone's file folders, select the recording, and send it securely via the Siilo app. It is also possible to record the learning moments, just like on WhatsApp, using the voice function button (microphone) in the chat itself. However, from our experience, this can result in incomplete recordings. Therefore, we would like to request you to use your phone's dictation function and send the recordings securely through the chat function. If you, as a participant, shared an audiotaped learning moment with the group chat, it will be acknowledged by the researchers within 24 hours by a message to thank you for recording.

**How to download the smartphone-application ‘Siilo’**

We kindly request you (participant) to download the Siilo application for free from the App Store or Google Play Store. Follow the steps below to download the Siilo application:

1. Download Siilo Messenger from the App Store or Google Play Store. Search for 'Siilo,' download the application, and start the registration process by opening the application.
2.
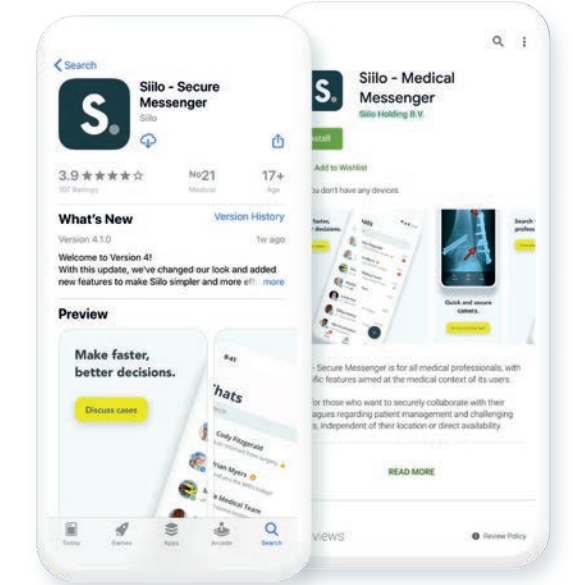
Complete the registration. Register your first and last name, profession, mobile number, and your (work) email address. Siilo will send a 6-digit confirmation code via SMS.
3. Grant permission to receive message notifications. There are two options for displaying notifications on your phone's home screen. If you choose 'Private,' you will only see a notification that you have received a new message. If you choose 'Easy,' you will see a preview of the content of the received message.
4. Set your PIN code and Touch/Face ID. Choose a 5-digit PIN code to access the application.

A more comprehensive guide to download the Siilo application can be found at the following link: <https://zel.nl/wp-content/uploads/2019/06/Handleiding-Siilo_A4-gecomprimeerd.pdf>
