# Supplementary material for: Unravelling the art of developing skilled communication: a longitudinal qualitative research study in general practice training
Source: Adv Health Sci Educ Theory Pract. 2024 Dec 17;30(4):1231–55. doi: 10.1007/s10459-024-10403-6 (PMC12391227; doi:10.1007/s10459-024-10403-6)
Supplement: Supplementary file 4 — Supplementary file4 (DOCX 19 KB) [file 10459_2024_10403_MOESM4_ESM.docx]

Supplementary information - Appendix D – Codebook

Article title: Unravelling the art of developing skilled communication: a longitudinal qualitative research study in General Practice training

Journal name: Advances in Health Sciences Education - Theory and Practice

Author names; Michelle Verheijden^1,2^; Angelique Timmerman1, Dorien de Buck, Anique de Bruin^2^, Valerie van den Eertwegh^2^, Sandra van Dulmen^3^, Geurt Essers, Cees van der Vleuten^2^, Esther Giroldi^1,2^.

Affiliation:

1. Care and Public Health Research Institute (CAPHRI)
2. School of Health Professions Education (SHE)
3. Netherlands Institute for Health Services Research, Utrecht, Netherlands (NIVEL)

E-mail address of corresponding author: [m.verheijden@maastrichtuniversity.nl](mailto:m.verheijden@maastrichtuniversity.nl)

| **Code** | | | **Description** | **Examples** | **Code used in following data-source (start-interviews, audio-diaries, exit-interviews)** |
| --- | --- | --- | --- | --- | --- |
| **Reason choice impactful experience** | | | *Reason why the trainee has chosen for a (specific) impactful experience related to learning communication as a starting point the stimulated-recall interview.* | *An experienced emotion like insecurity during or after an encounter.* | Start interviews and exit interviews |
| **Stimulus and-or trigger of impactful experience** | | | An impactful experience that triggers the trainee to start learning. Or, the reason why the GP trainee has chosen for a (specific) impactful experience related to communication behaviour. | *An experienced emotion like uneasiness or received feedback from supervisor. Moreover, experienced challenges during an encounter like having a disagreement with a patient about therapy can trigger trainees to start learning.* | Start interviews, audio diaries, exit interviews |
| **Learning strategies** | | | Actions initiated by an actor (e.g. trainee, supervisor, peer) used to induce learning of communication. | *Trainees ask specific feedback or watch a videotaped consultation with supervisors.* | Start interviews, audio diaries, exit interviews |
| **Awareness about own communication behaviour** | | Becoming aware of communication behaviour | *By discussing videotaped consultations with supervisors, trainees become aware of own behaviour. Also, reflection after clinic can stimulate trainees’ awareness, which may happen at a time when there is mental space for the trainee to reflect and think about the encounter* | Start interviews, audio diaries, exit interviews |  |
| Self-observation* | Recognizing trainees’ own thoughts or communication behaviours | *By reviewing video-taped consultations, trainees become aware of own behaviour. For example, trainees notice they cross their arms a lot during an encounter.* | Start interviews, audio diaries, exit interviews |  |  |
| Self-judgment* | Grading appropriateness of the trainees’ communication behaviours | *By reviewing videotaped-consultations, trainees are able to recognise inappropriate postures such as crossed arms* | Start interviews, audio diaries, exit interviews |  |  |
| Self-reaction* | Adapting or implementing change in trainees’ communication behaviours | *Trainees adapted their communication behaviour by reminding themselves to adopt an open posture* | Start interviews, audio diaries, exit interviews |  |  |
| Self-monitoring* | An in-the-moment self-awareness of trainees’ communication behaviour. | *During an encounter, trainees asked questions to themselves, such as: Did my communication behaviour turned out the way I wanted to be?’* | Start interviews, audio diaries, exit interviews |  |  |
| Self-assessment* | Evaluating communication behaviour without focusing on a specific communication behaviour at a specific moment in time, often leading to general reflections about multiple applied communication behaviour | *Trainees reflecting on their applied communication behaviour, almost a week, after clinic, yielding a generalised evaluation of their communication behaviour* | Start interviews, audio diaries, exit interviews |  |  |
| **Identifying alternative communication behaviour** | | Identifying alternative communication behaviours | *Through learning conversations with supervisors, trainees identify through these conversations concrete sentences from supervisor to adjust their communication behaviour* | Start interviews, audio diaries, exit interviews |  |
| **Experimenting new communication behaviour** | | Experimenting with acquired new communication behaviours | *Experimenting usually with new communication behaviour. For example, improve emotional reflections by explicitly naming what the trainee sees to provide insight for both trainee and patient.* | Start interviews, audio diaries, exit interviews |  |
| **Evaluating new communication behaviour** | | Evaluating whether new communication behaviour was or turned out to be effective or not. | *Patient’s feedback seemed an important source guiding trainees in evaluating alternative new communication behaviour, for example based on patient’s response like expressed gratitude.* | Start interviews, audio diaries, exit interviews |  |
| **Write down (learning goals)** | | Writing down learning goals as a tool to memorize. | *By writing down alternative communication behaviours trainees can more easily memorize it for experimenting* | Start interviews, audio diaries, exit interviews |  |
| **Conditions supporting and or inhibiting learning of communication** | | | Conditions that have a supporting and/or inhibiting effect/impact on learning communication of the trainee. In other words, these include requirements for learning and are not initiated by an actor to support and/or inhibit learning. | *Conditions supporting and/or inhibiting learning. For example, trainees feel the lack time in clinical practice to spend for learning e.g., preparing for training sessions* | Start interviews and exit interviews |
| **Evaluation of personal development** | | | The GP trainee monitors and evaluates knowledge, feelings, and thoughts during the process of learning communication. | *Development of a specific communication learning theme like setting boundaries throughout trainees’ training.* | Start interviews and exit interviews |
| **Internalising communication behaviour** | | | New communication behaviour becomes an integrated part of the personal repertoire of the trainee. | *Feelings like automaticity indicating that new communication became part of communication repertoire and trainee is able to choose between different, new, communication behaviours from personal repertoire.* | Start interviews and exit interviews |
| **Reflection on past performance (applied communication behaviour)** | | | During recall of performed communication behaviour, the trainee elaborates and reflects upon communication behaviour | *Emotions, feelings, or concrete communication behaviours are described when reflecting on communication behaviour* | Start interviews, audio diaries, exit interviews |
| **Personal learning goals** | | | *Trainee sets personal learning goals* | *For example, setting boundaries, exploring reasons for encounter or dealing with long-winded patients* | Start interviews, audio diaries, exit interviews |
| **Behavioural intentions for future performance or learning** | | | Trainee describes how he or she could perform communication behaviour in future clinical encounters. | *For example, stating concrete sentences how to set boundaries to a long-winded patient in future clinical encounters.* | Start interviews, audio diaries, exit interviews |
| **Outcome of stimulated recall interview** | | | Trainee describes the general outcome including insights after or during the simulated recall reflection. This is the overall ending question. | *What the trainee takes out of the stimulated-recall reflection, such as conclusions, feelings, thoughts, and acquired knowledge about applied communication behaviour* | Start interviews and exit interviews |
| **Outcome of audio-recorded learning moment** | | | What the trainee takes out of the recorded learning moment in general. | *What the trainee takes out of the learning moment can be very broad, ranging from raised awareness to an intention for learning to obtained communication behaviour to a learning goal.* | Audio diaries |

**Definitions based on Johnson et al. 2023 and tailored to the context of this study.*
