# Supplementary material for: Unravelling the art of developing skilled communication: a longitudinal qualitative research study in general practice training
Source: Adv Health Sci Educ Theory Pract. 2024 Dec 17;30(4):1231–55. doi: 10.1007/s10459-024-10403-6 (PMC12391227; doi:10.1007/s10459-024-10403-6)
Supplement: Supplementary file 5 — Supplementary file5 (DOCX 15 KB) [file 10459_2024_10403_MOESM5_ESM.docx]

Supplementary information - Appendix E – Demographic characteristics of participants

Article title: Unravelling the art of developing skilled communication: a longitudinal qualitative research study in General Practice training

Journal name: Advances in Health Sciences Education - Theory and Practice

Author names; Michelle Verheijden^1,2^; Angelique Timmerman1, Dorien de Buck, Anique de Bruin^2^, Valerie van den Eertwegh^2^, Sandra van Dulmen^3^, Geurt Essers, Cees van der Vleuten^2^, Esther Giroldi^1,2^.

Affiliation:

1. Care and Public Health Research Institute (CAPHRI)
2. School of Health Professions Education (SHE)
3. Netherlands Institute for Health Services Research, Utrecht, Netherlands (NIVEL)

E-mail address of corresponding author: [m.verheijden@maastrichtuniversity.nl](mailto:m.verheijden@maastrichtuniversity.nl)

| **Pseudonym** | **Gender** | **Age** | **Training year** |
| --- | --- | --- | --- |
| 1002 | F | 32 | 1 |
| 1003 | F | 30 | 1 |
| 1004 | F | 31 | 3 |
| 1005 | F | 32 | 3 |
| 1006 | M | 30 | 1 |
| 1007 | M | 28 | 1 |
| 1008 | F | 35 | 3 |
| 1009 | M | 31 | 3 |
| 1010 | F | 30 | 1 |
| 1011 | M | 30 | 3 |
| 1012 | F | 28 | 1 |
| 1013 | F | 28 | 1 |
| 1014 | F | 28 | 1 |
