# Supplementary material for: Unravelling the art of developing skilled communication: a longitudinal qualitative research study in general practice training
Source: Adv Health Sci Educ Theory Pract. 2024 Dec 17;30(4):1231–55. doi: 10.1007/s10459-024-10403-6 (PMC12391227; doi:10.1007/s10459-024-10403-6)
Supplement: Supplementary file 6 — Supplementary file6 (DOCX 16 KB) [file 10459_2024_10403_MOESM6_ESM.docx]

Supplementary information - Appendix F – Definitions terms used in the six-stage conceptual learning model

Article title: Unravelling the art of developing skilled communication: a longitudinal qualitative research study in General Practice training

Journal name: Advances in Health Sciences Education - Theory and Practice

Author names; Michelle Verheijden^1,2^; Angelique Timmerman1, Dorien de Buck, Anique de Bruin^2^, Valerie van den Eertwegh^2^, Sandra van Dulmen^3^, Geurt Essers, Cees van der Vleuten^2^, Esther Giroldi^1,2^.

Affiliation:

1. Care and Public Health Research Institute (CAPHRI)
2. School of Health Professions Education (SHE)
3. Netherlands Institute for Health Services Research, Utrecht, Netherlands (NIVEL)

E-mail address of corresponding author: [m.verheijden@maastrichtuniversity.nl](mailto:m.verheijden@maastrichtuniversity.nl)

| **Stages** *(in the blue boxes)* | | |
| --- | --- | --- |
| Triggering | *Impactful experience that triggers learning* |  |
| Becoming aware | *Becoming aware of communication behaviour* |  |
| Identifying | *Identifying alternative communication behaviour* |  |
| Experimenting | *Experimenting with new communication behaviour.* |  |
| Evaluating | *Evaluating whether new communication behaviour was effective.* |  |
| Internalising | *Internalising new communication behaviour into personal repertoire* |  |
| **Conditions** *(in italics)* | | |
| - a constructive relationship with supervisors - a nurturing workplace - alignment in content between training and practice - narrative feedback from formative assessments - time for mandatory reflections. | *Conditions stimulating trainees’ learning process of communication, including requirements for learning that are not initiated by an actor.* |  |
